# Supplementary material for: Type IV Collagen in Human Colorectal Liver Metastases—Cellular Origin and a Circulating Biomarker
Source: Cancers (Basel). 2022 Jul 13;14(14):3396. doi: 10.3390/cancers14143396 (PMC9325127; doi:10.3390/cancers14143396)
Supplement: Supplementary file 1 [file cancers-14-03396-s001.zip › cancers-1754876-supplementary.pdf]

Supplementary Materials

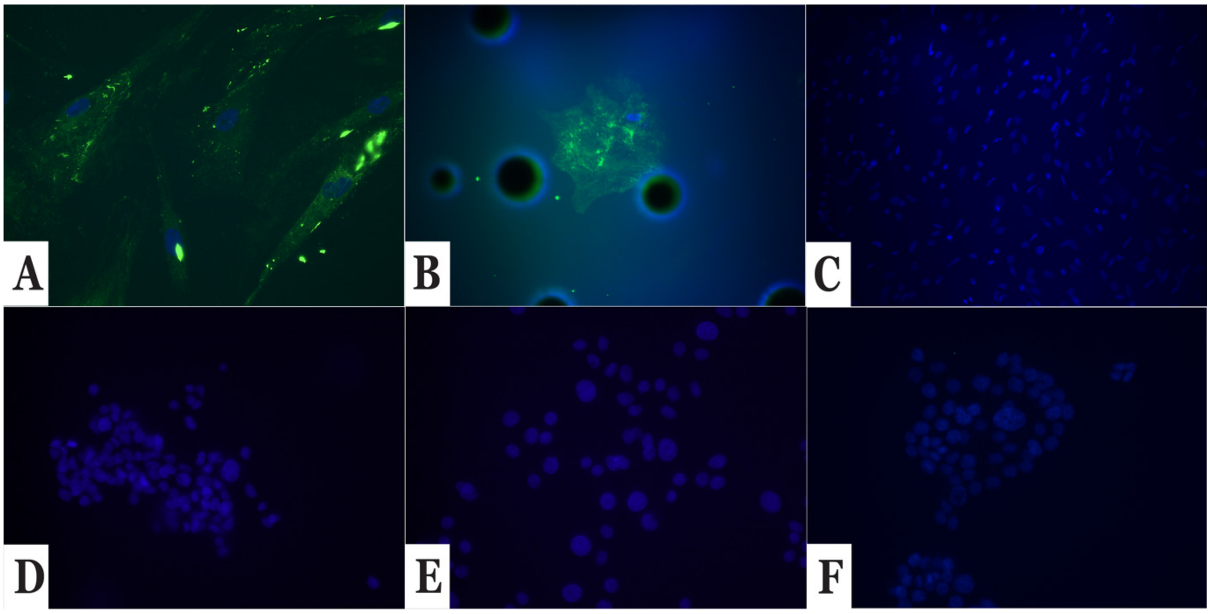

Supplementary Figure S1. Immunofluorescence of COL IV (green) in fibroblasts and cancer cell lines. (**A**) Immunofluorescence of CCD-18Co fibroblasts showing collagen IV expression (green). (**B**) Immunofluorescence of CCD-112CoN fibroblasts showing collagen IV (green). No collagen IV expression was observed in (**C**) WS1 fibroblast, (**D**) LoVo cancer cells, (**E**) SW480 cancer cells, and (**F**) HT-29 cancer cells. (**A, B, C**) magnification ×20 and (**D, E, F**) ×40. Nuclear staining with DAPI (blue).

Supplementary Table S1. Prognostic factors and correlation to cCOL IV in CLM patients

**Variable**	***p*-value**	**Number of patients with available data**	**Statistical analysis**
Size of largest metastases (mm)	**0.0338**	138	Pearson's correlation
Interval CRC-CLM (months)	0.0697	132	Pearson's correlation
Age at surgery/blood sample	0.1311	138	Pearson's correlation
Number of metastases	0.3625	138	Pearson's correlation
Preoperative chemotherapy^ (yes vs. no)	**0.0023**	135	Independent t-test
TNM-stage (I–III vs. IV)	**0.0377**	133	Independent t-test
Largest metastases >5 cm (yes vs. no)	0.1040	138	Independent t-test
More than 1 metastases (yes/no)	0.1280	138	Independent t-test
Interval CRC–CLM >12 months (yes vs. no)	0.3716	138	Independent t-test
Age over 70 y/o at surgery (yes vs. no)	0.3946	138	Independent t-test
N-positive primary tumor (yes vs. no)	0.4182	131	Independent t-test
Rectum vs. colon cancer	0.7588	138	Independent t-test
Female vs. Male	0.8434	138	Independent t-test
Primary tumor left at sampling (yes vs. no)	0.9764	138	Independent t-test
Extrahepatic disease (yes vs. no)	0.9767	138	Independent t-test

CRC: colorectal cancer, CLM: colorectal liver metastases, mm: millimeter, ^Neoadjuvant + Conversion therapy
